# Evaluation of within-host evolution of methicillin-resistant *Staphylococcus aureus* (MRSA) by comparing cgMLST and SNP analysis approaches

**DOI:** 10.1038/s41598-022-14640-w

**Published:** 2022-06-22

**Authors:** Amaya Campillay Lagos, Martin Sundqvist, Fredrik Dyrkell, Marc Stegger, Bo Söderquist, Paula Mölling

**Affiliations:** 1 Department of Laboratory Medicine, Clinical Microbiology, Faculty of Medicine and Health, Örebro University, Örebro, Sweden; 2 1928 Diagnostics, Gothenburg, Sweden; 3 Department of Bacteria, Parasites and Fungi, Statens Serum Institut, Copenhagen, Denmark

**Keywords:** Clinical microbiology, Infectious-disease diagnostics, Microbial genetics

## Abstract

Whole genome sequencing (WGS) of methicillin-resistant *Staphylococcus aureus* (MRSA) provides high-resolution typing, facilitating surveillance and outbreak investigations. The aim of this study was to evaluate the genomic variation rate in MRSA, by comparing commonly used core genome multilocus sequencing (cgMLST) against single nucleotide polymorphism (SNP) analyses. WGS was performed on 95 MRSA isolates, collected from 20 carriers during years 2003–2019. To assess variation and methodological-related differences, two different cgMLST schemes were obtained using Ridom SeqSphere+ and the cloud-based 1928 platform. In addition, two SNP methods, 1928 platform and Northern Arizona SNP Pipeline (NASP) were used. The cgMLST using Ridom SeqSphere+ and 1928 showed a median of 5.0 and 2.0 allele variants/year, respectively. In the SNP analysis, performed with two reference genomes COL and Newman, 1928 showed a median of 13 and 24 SNPs (including presumed recombination) and 3.8 respectively 4.0 SNPs (without recombination) per individual/year. Accordantly, NASP showed a median of 5.5 and 5.8 SNPs per individual/year. In conclusion, an estimated genomic variation rate of 2.0–5.8 genetic events per year (without recombination), is suggested as a general guideline to be used at clinical laboratories for surveillance and outbreak investigations independently of analysis approach used.

## Introduction

*Staphylococcus aureus* is a commensal that can be found in the human microbiota of the nasal mucosa in 20–40% of the population^1^. However, this bacterium is also associated with infections in humans, ranging from mild superficial skin and soft tissue infections, to severe conditions such as infective endocarditis, bacteraemia, osteomyelitis and septic arthritis. In Sweden 3112 cases of new MRSA episodes were reported in 2020, a decrease of 19% compared to 2019 before the Covid-19 pandemic^2^. Previously hospital-acquired MRSA (HA-MRSA) was mainly a healthcare-related problem, today, community-acquired MRSA (CA-MRSA) appears to be the main route of transmission. CA-MRSA lineages have over time been observed in healthcare-related outbreaks, therefore the distinction between the two groups has become blurred^3,4^. Since the year of 2000, MRSA is included in the Public Health Agency of Sweden’s (PHAS) monitoring program as a notifiable disease where contact tracing is mandatory regarding every case according to the Communicable Diseases Act. For every new MRSA case, the local infection control unit decides to whether or not eradicate the bacteria. MRSA carriers are then followed with sampling once a year. For surveillance, basic molecular typing that divides isolates into clonal complexes (CC) based on multilocus sequence typing (MLST) as well as *Staphylococcus aureus* protein A (*spa*) typing is generally used^5,6^. To obtain more detailed information, typing of MRSA by whole genome sequencing (WGS) is nowadays widely used to get a more extensive surveillance, e.g. in an outbreak situation^7,8^.

WGS provides access to complete microbial genomes, and its discriminatory power enables to perform large-scale genomic epidemiological studies on a national level^9^.

The use of WGS facilitates identification of emerging lineages within the community and/or responsible for hospital outbreaks due to the continuous alteration of the genome that occurs through replication errors, acquisition of genetic material from other bacteria by horizontal gene transfer of mobile genetic elements and recombination events^3,10^. There are several widely used approaches for depicting the relatedness of isolates using genome data, such as core genome multilocus sequence typing (cgMLST) or single nucleotide polymorphism (SNP) analyses. *S. aureus* cgMLST enables a standardized, discriminatory and reproducible method using the same defined core genome for the differentiation of MRSA and produces a definitive nomenclature to be shared between different stakeholders if the same cgMLST scheme is used^11^. SNP-based analysis, on the other hand, often provides an even higher discriminatory power, but comparison between laboratories is more difficult due to the variability in settings and use of reference genomes. The threshold settings and the variety of SNP filters needs to be the same if comparison between laboratories is to be accomplished^10–13^. *S. aureus* evolves primarily through replication-induced point mutations, which accumulate SNPs over time. There are few reports in this area, mainly focusing on short-term studies about small outbreaks in care facilities or regarding evolution within a specific MLST or CC. According to these studies, the estimated mutation rate for MRSA varied between 2 and 10 SNPs in a single genome per year^10,14–16^. However, the mutation rate can be defined by single nucleotide alterations in a SNP analysis but it can also be a genetic variation as a single SNP, several SNPs or allelic variations in cgMLST and hereby referred as the genomic variation rate in MRSA carriers^11^.

In the present study, the aim was to evaluate the genomic variation rate in MRSA using isolates from long-term carriers by comparing two commonly used analysis approaches i.e. cgMLST and SNP as guidelines for clinical laboratories in surveillance and outbreak investigations.

## Results

### Molecular typing of MRSA in long-term carriers

All MRSA isolates (n = 95) were successfully whole genome sequenced and subsequently analysed. Four samples, two from patient 5, one from patient 9 and one from patient 12 were excluded due to change of sequence type (ST) and complex type (CT), showing an obvious acquisition of a different MRSA strain during the follow up as shown in Table 1. The remaining samples (n = 9) for these patients were included for detailed analyses.

**Table 1:**
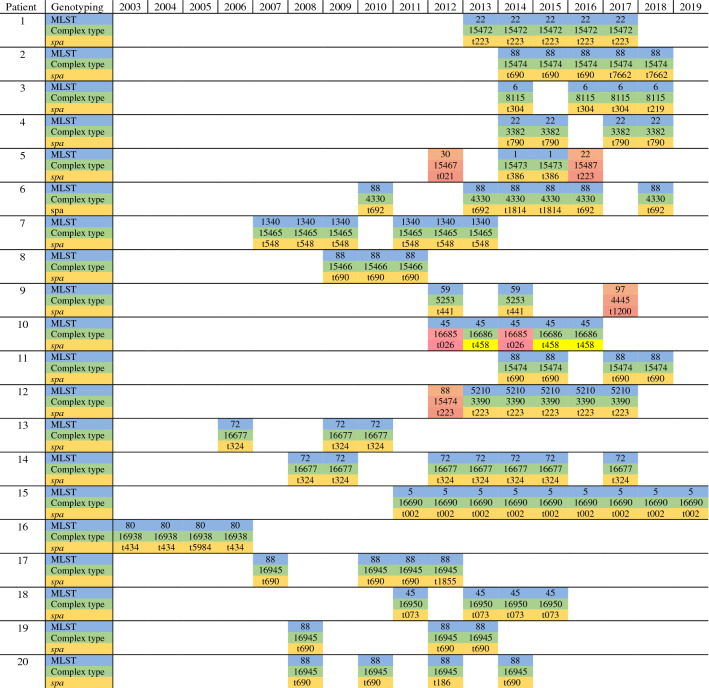
Classification of samples according to its multilocus sequencing type (MLST), core genome MLST (Complex type) and *Staphylococcus aureus* protein A (*spa*) per patient and year performed with SeqSphere+.

The change of MRSA strain appears clearly (orange) for patients 5, 9 and 12 therefore the samples (n = 4) were excluded from the calculations. Patient 10 has two of five samples with a different CT- and *spa*-type consequently, patient 10 was divided in two groups, patient 10.1 (dark colour) and patient 10.2 (light colour). Three samples (light yellow) were re-analyse for *spa* with Sanger sequencing due to failure with WGS to define *spa*. In addition, five more samples; two samples from patient 2, two samples from patient 6 and one sample from patient 16 were also sequenced with Sanger to verify the results obtained with WGS due to change of *spa* type within the patient during the follow up.

Eleven different MLST-types were identified among the carriers where the majority belonged to ST88 (7 patients/29 samples), ST22 (2/9), ST6 (1/4), ST1 (1/2), ST1340 (1/6), ST59 (1/2), ST45 (2/9), ST5210 (1/5), ST72 (2/10), ST5 (1/9) and ST80 (1/4). Eight different CC were found among the patients, CC1 (1 patient/2 samples), CC5 (3/19), CC8 (2/10), CC22 (3/14), CC45 (2/9), CC59 (1/2), CC80 (1/4) and CC88 (7/29). The cgMLST analysis run by Ridom SeqSphere+ further divided the samples into 17 different CT groups as shown in Table 1.

Twenty-one different *spa* types were found in the collection. Eight samples were re-analysed with Sanger sequencing, of these samples, five were re-run to verify a change of *spa* within a patient during the follow up. The remaining three samples were re-run due to failure with WGS to define a *spa* type but successfully analysed with Sanger sequencing.

Two different CT and *spa* types were present in patient 10, therefore, this patient’s samples were divided in two groups, 10.1 and 10.2 as shown in Table 1.

**Figure 1:**
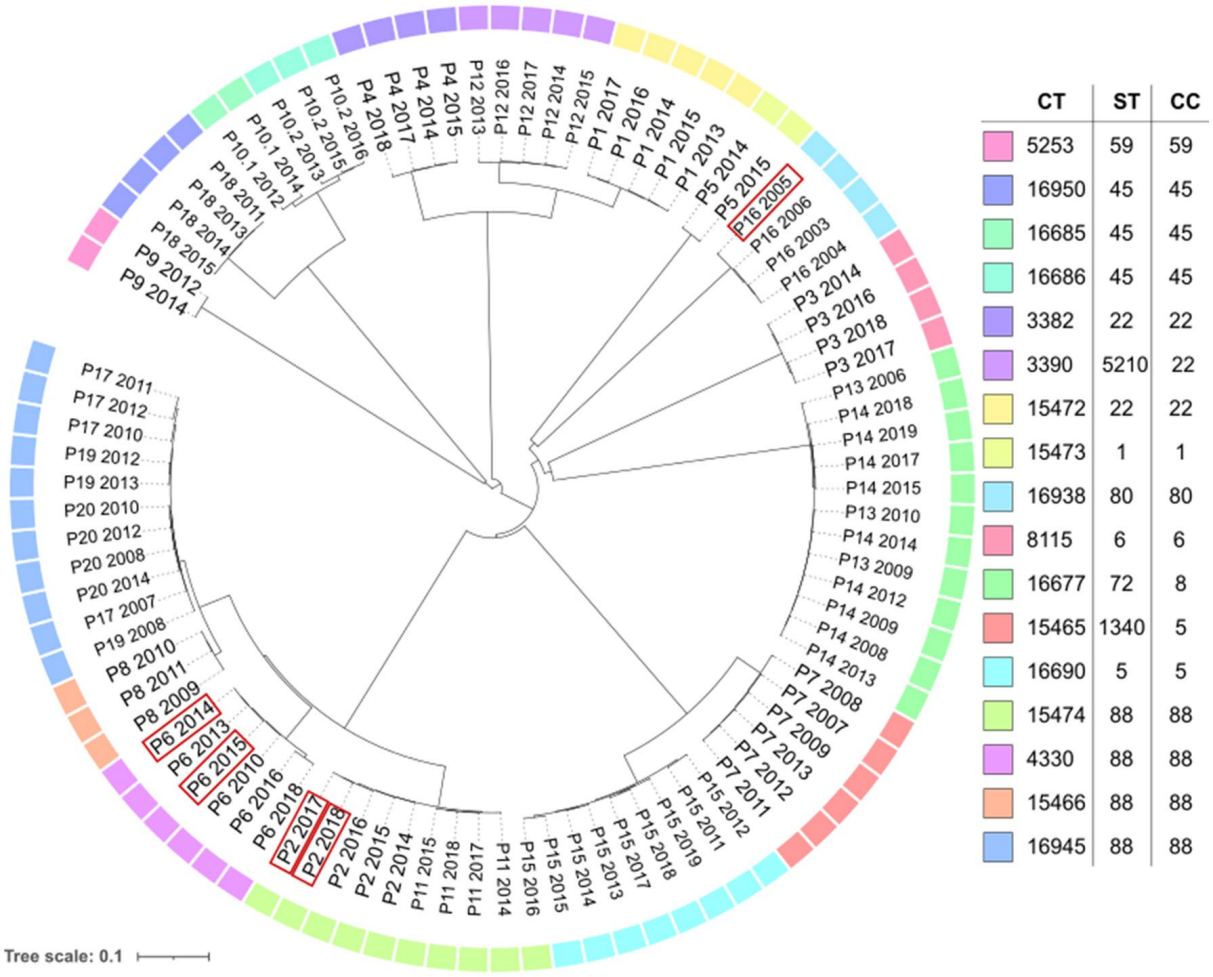
Circular neighbour joining tree made based on the data analysed with SeqSphere+ where missing pairwise values were ignored. The tree is arranged according to the MRSA samples coloured by the division into a complex type (CT) based on the core genome multilocus sequence types (cgMLST) analysis with SeqSphere+. MLST sequence type (ST) and Clonal complex (CC) are also shown in the figure. The remaining 91 samples included in the analyzes are shown in the phylogenetic tree and the samples where the *spa* type differed are marked in a red box.

The relatedness of the isolates included in the analysis is shown in the phylogenetic tree generated from the CT analysis performed with Ridom SeqSphere+ in Fig. 1.

### Comparisons of within-host evolution by cgMLST approaches in MRSA carriers

The cgMLST analysis using Ridom SeqSphere+ showed a median of 5.0 (range 0.5–10.8) allele variants/year per individual while the analysis by 1928 displayed a median of 2.0 (range 0.5–6.8) allele variants/year. The summary of the estimated genomic variation rate for each patient and method are shown in Table 2.

**Table 2:** Calculation of the mean distance between MRSA-samples for each patient and for the different analysis performed; where the cgMLST was calculated according to the pairwise allelic differences between samples and the SNP according to the pairwise SNP differences between samples.

Patient	Samples	Year^A^	cgMLST^B^		SNP NASP^C^		SNP with recombination 1928		SNP without recombination 1928	
			SeqSphere COL	1928 Newman	COL	Newman	COL	Newman	COL	Newman
1	5	4	5.8	1.5	5.8	5.8	47.0	22.3	5.5	5.0
2	5	4	6.5	4.0	8.0	8.0	25.0	13.5	6.5	5.8
3	4	4	9.3	6.5	9.3	9.0	36.0	13.3	6.3	6.3
4	4	4	8.3	6.8	9.3	9.3	41.3	13	4.8	4.8
5	2	3	1.5	0.5	2.5	2.0	10.8	3.8	1.0	1.0
6	6	8	4.0	2.3	4.8	4.8	14.1	10.9	3.4	3.5
7	6	6	10.8	3.5	12.5	12.3	32.5	17.8	8.7	8.8
8	3	2	6.5	4.5	8.0	8.0	12.0	20	6.5	6.0
9	2	2	0.5	0.6	2.2	1.2	9.4	3.6	1.4	1.2
10.1	2	2	5.5	1.5	6.5	5.5	37.0	13.0	1.0	1.0
10.2	3	3	4.3	2.7	5.7	4.3	26.0	19.3	2.0	2.0
11	4	4	4.0	1.5	5.0	5.0	29.0	17.5	3.8	3.8
12	5	5	6.0	3.8	6.8	6.6	23.6	13.2	4.4	4.0
13	3	4	4.5	2.0	4.8	4.8	28.3	6.8	3.5	3.5
14	9	11	2.7	1.1	2.5	2.5	16.1	4.1	2.0	1.7
15	9	8	6.3	3.4	6.3	6.3	23.9	10.1	4.3	4.0
16	4	3	9.3	4.0	10.3	8.3	14.7	14.3	7.7	7.7
17	4	5	5.0	1.2	6.4	6.2	8.4	9.4	4.8	5.0
18	4	4	2.3	1.3	3.5	2.3	15.5	13.3	0.75	0.8
19	3	5	1.6	0.6	2.6	2.6	12.8	6.2	1.2	1.2
20	4	6	4.2	1.0	5.2	5.2	12.8	11.3	3.3	2.3
Mean			5.2	2.6	6.1	5.7	22.7	12.2	4.0	3.8
Median			5.0	2.0	5.8	5.5	23.6	13.0	4.0	3.8
Standard deviation			2.7	1.8	2.8	2.8	11.3	5.3	2.0	2.3

A: years passed from the first to the last sample collection. B: cgMLST = alleles per year. C: SNP = SNPs per year.

### Comparison of within-host evolution by SNP approaches

The 1928 SNP analysis, excluding recombination events, showed a median of 4.0 (range 0.75–8.7) and 3.8 (range 0.8–8.8) SNP changes per individual/year whilst the analysis, including recombination events, showed a median of 23.6 (range 8.4–47.0) and 13.0 (range 3.6–22.3) using the COL- and Newman references, respectively. The considered percentage of the genome compared to the reference ranged from 85.4 to 92.4% for the COL reference and 82.6% to 91.3% for the Newman reference. The corresponding results for the NASP pipeline were 5.8 (range 2.2–12.5) and 5.5 (range 1.2–12.3) changes per individual/year (Table 2) were the considered percentage of the genome compared to the reference was 80.53% for the COL reference and 77.98% for the Newman reference.

### Comparison of estimated genomic variation rate according to ST-type

**Figure 2:**
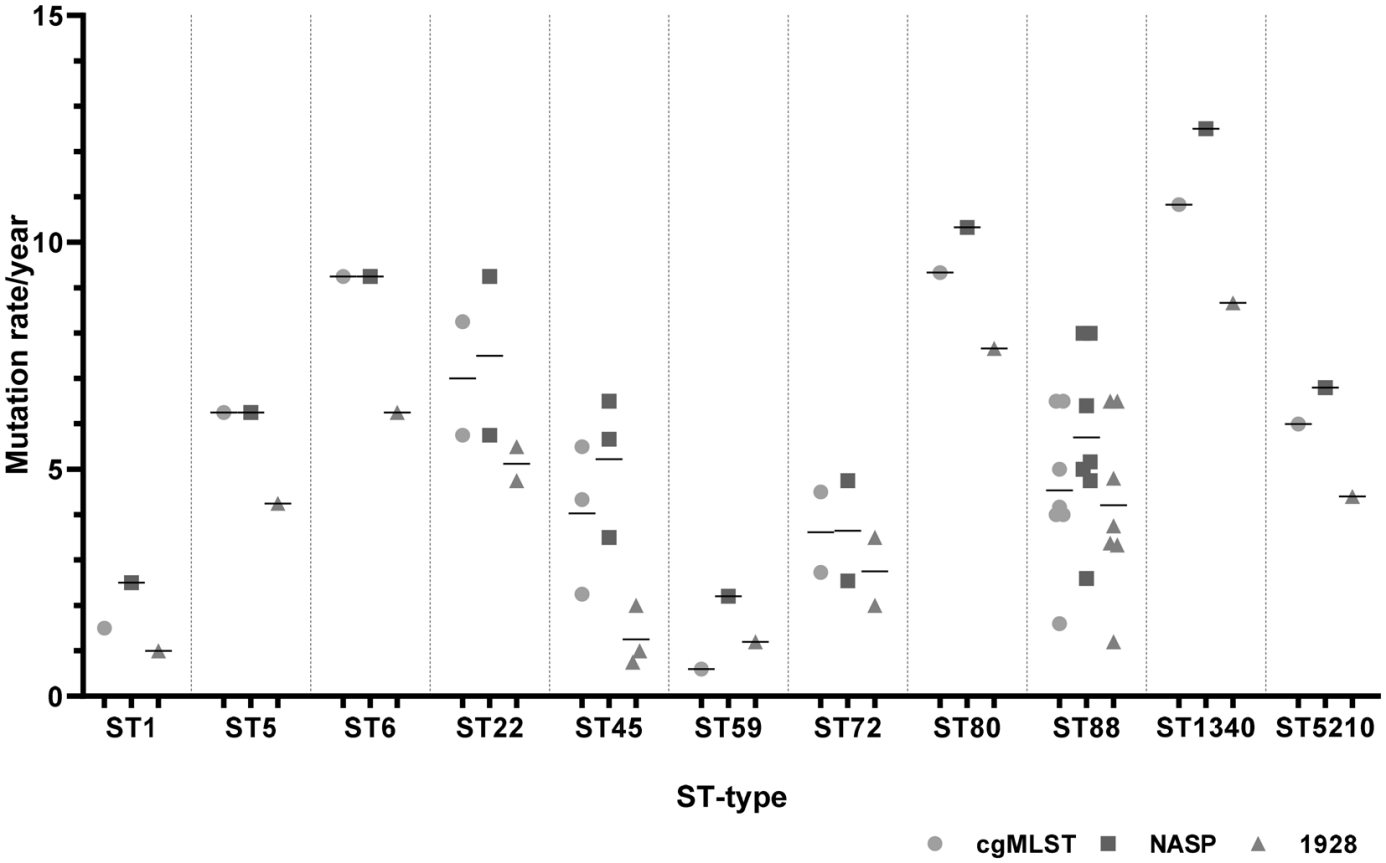
The mean for the genomic variation rate per year for all the ST found among the 20 MRSA-carriers are shown in the dot plot analysis. The cgMLST analysis (circle) was performed with SeqSphere+. Two different SNP-analysis were performed, the NASP-pipeline (square) and the cloud based software 1928 (triangle). The plot is based according to the results obtained using the COL reference. The three analysis have been grouped after each ST to facilitate visual comparison of the mean between them. The 1928 analysis show deviating/lower results compared to the results obtained with the cgMLST- and NASP-analysis where the results are similar. ST88, which is the ST with most samples, shows that the cgMLST and 1928 analysis are more comparable with each other than with NASP. A larger collection could give different results to the one observed in this study.

Our results showed a genomic variation rate between the eleven different STs found. The dominant clone ST88 showed an estimated genomic variation rate of 4.5 alleles/year, 5.6 SNP/year (NASP) and 4.1 SNP/year (1928 WOR). The ST1 (n = 2) and ST1340 (n = 6) showed the lowest and highest estimated genomic variation rate of 1.5 and 10.8 alleles/year and 2.5 and 12.5 SNP/year (NASP) whereas the rate for 1928 (WOR) was 1.0 and 8.7 SNP/year, respectively. The estimated genomic variation rate for each type was calculated by adding the genomic variation rate for each of the STs divided with the total number of years among the patients within the same ST as shown in Table 3 and graphically in Fig. 2.

**Table 3:** Estimated genomic variation rate within each sequence type (ST) based only on the COL reference sequence.

ST type	Patients/samples	SeqSphere+ alleles/year	NASP SNPs/year	1928 WOR^A^ SNPs/year
1	1/2	1.5	2.5	1.0
5	1/9	6.2	6.3	4.2
6	1/4	9.2	9.2	6.2
22	2/9	6.9	7.5	5.2
45	2/9	3.9	5.2	1.2
59	1/2	0.6	2.2	1.2
72	2/10	3.6	3.6	2.8
80	1/4	9.2	10.3	7.6
88	7/29	4.5	5.6	4.1
1340	1/6	10.8	12.5	8.75
5210	1/5	5.9	6.8	4.4

A: WOR = without recombination.

## Discussion

For correct interpretation of temporal divergence of bacterial lineages of SNPs and allele differences, knowledge about the genomic variation rate of MRSA is critical. Molecular methods such as typing of the bacteria are used to confirm if a new case of MRSA belongs to a known clone or if it is unique. Surveillance of MRSA is essential in order to identify modes and routes and to prevent or limit further transmission. Nowadays many laboratories perform WGS, a technique that more commonly replace *spa* typing as the golden standard for surveillance and outbreak investigations^14,15^.

The continuous development of high-throughput measurement techniques as WGS produces large numbers of data sets therefore access to bioinformatics knowledge is the key in rapidly computing and interpreting the information obtained from the analysis. Hence, more user-friendly bioinformatics platforms need to be developed to allow easier interpretation, management and exploration of data for personnel who do not have bioinformatics expertise.

In the present study, we describe how different ways to analyse WGS data for MRSA using both SNP and cgMLST affects the estimated genomic variation rate of MRSA within carriers. WGS combined with user-friendly software, such as SeqSphere+, cloud-based pipelines like 1928 and bioinformatics tools including NASP, reveals more detailed genetic information than classical typing technologies. Genetic mutations in organisms causes diversity and can be beneficial, harmful, or neutral, depending on their context or location. Previous studies have used mutation rate as a marker for relatedness between isolates during outbreaks and for patient-to-patient transmission and have demonstrated that the estimated mutation rate of MRSA can range from 2–10 SNP/year^10,14–17^. In this study the estimated genomic variation rate was found to be within a range of 2.0–5.0 alleles/year with the cgMLST analysis, 3.8–5.8 SNP/year in the SNP-analysis (excluding recombination) and 13 and 23.6 SNP/year (including recombination) depending on the method and the reference used. Diversification of MRSA strains occurred in three patients during the follow-up (Table 1) and consequently four samples were excluded from the overall calculations: two samples from patient 5, one from patient 9 and one from patient 12. The detection of another MRSA strain can be explained with intra-host diversification where the bacterial strain can differ between infection and colonising sites so both lineages can be carried at all-time points or colonisation from a close relative^18–20^. Overall, the results showed that the genomic variation rate does not differ much between the methods used if recombination events are not included in the analysis as shown in Table 2. Recombination events, however, are handled differently by cgMLST if compared to a SNP alignment, as recombinant regions with a high density of SNPs can be filtered, while cgMLST methods will collapse these regions into a smaller number of allelic changes^11^. Although the collection of samples for making a more categorized estimation according to the different STs is small, our results are comparable with the results obtained in previous studies and we can see that the genomic variation rate seems to vary within the different STs^10,14–17^.

The majority of samples belonged to the group of CA-MRSA, which are known for being more virulent and transmissible than HA-MRSA causing infection even in healthy individuals^21^. CA-MRSA was defined through identification of MRSA and fulfilled the following criteria; outpatient care, positive within 48 h of admission to a hospital, no medical history of MRSA infection or colonisation, among others^22^. The CA-MRSA clone ST88 was found to be the dominant clone in our collection (n = 29). ST88 has been found in many countries including Sweden and is the predominant clone in Sub-Saharan Africa^23^. In our collection, ST88 showed an estimated genomic variation rate that fits into the estimated genomic variation rate for the whole collection, which is not that surprising due to it is a quite large proportion of the collection. The estimated genomic variation rate however, varied between the other STs. In the present study, *spa* typing was performed with SeqSphere+ using WGS data, however, the use of WGS did not succeed recognising one of the *spa* types. Therefore, *spa* typing with Sanger sequencing was performed for the isolates where the *spa* type had not succeeded and additionally in the cases where only the *spa* type differed (same ST and CT) in isolates from the same patient. Sanger sequencing verified this change of *spa* types to be correct and the isolate that failed was found to be t458. The *spa* type t458 surprisingly contained only one repeat, which indicates fail in the data analysis and not the WGS, since short read WGS is known for having difficulties to analyse *spa* types with many similar repeats^24^. In our laboratory, we use WGS for routine typing and surveillance of MRSA. Here we also included the *spa* type due to many laboratories still use *spa* typing as standard method for typing of MRSA even though it lacks of discrimination power in outbreak investigations^25–27^.

The selection of a reference genome with a well-defined core genome can be a source of bias if it is not closely related to the analysed sequences and can affect the subsequent analyses such as the detection of genetic events^28^. This could explain why the cgMLST analysis performed with 1928 showed a lower estimated genomic variation rate compared to SeqSphere+ since they are based on two different references, i.e. Newman and COL. The core genome from the Newman’s reference is only based on 1704 genes compared to the COL reference genome that is 1861 genes resulting in a difference of 157 genes, which could support the variation of random genetic variations. In contrast to the cgMLST results based on different core genomes, the SNP-based analyses performed with NASP and 1928 actually showed comparable results, independently of the reference genome used. Otherwise, it is usually more difficult to compare results from SNPs analyses when using different references since anything that does not match the reference will be excluded from the results^11^. Therefore, the choice of reference genome in a SNP analysis is known to be of high importance and needs to be very closely related to the isolates if high resolution is the goal, as in outbreak investigations.

A few limitations need to be considered in our study. The genomic variation rate per ST was calculated using a small collection of samples. However, our results indicate a variation of genomic variation rate between different STs, therefore, it is important to investigate a larger sample collection since different STs dominate in different parts of the world. In addition, in this study, the mutation rate was studied within-host and not between carriers, which often is the case during an outbreak investigation. Another limitation is that in the clinical laboratory, only one random colony is chosen to analyse further and all the molecular results are thus based on this colony. This might have led to a selection of the more dominant strain during a certain period of time although several strains might have been present in the patient’s flora. Therefore, this could have affected the presence of “new” strains in some patients. Another possibility is that the patient did acquire a new strain through infection from another person or that the carrier possesses a mixed MRSA-population that is (occasionally) more predominant^18–20^. One important aspect to consider about NASP is that it does not actively consider recombinant sites. To take recombination events into account the data requires further analyses, which was not done since few SNPs between person-isolates were observed.

In conclusion, the result of this study suggest that an estimated genomic variation rate of 2.0–5.8 (allele or SNP) per year can be used as a guideline in clinical laboratories for surveillance and outbreak investigations, regardless of the analysis method used i.e. cgMLST or SNP if excluding recombination events. However, it should be taken into account that the estimated genomic variation rate may vary in different MLST sequence types but also the choice of reference genome and typing method used due to the overall variability and suitability they possess.

## Materials and methods

### Ethical considerations

In the present study only subcultured bacterial isolates that were previously collected and retrieved, as part of the Swedish monitoring program, was stored. No tissue material or other biological material from patients was stored. No patient information was collected. Thus, the requirement for patient consent was waived and therefore ethical approval was not required.

### Sample selection

**Figure 3:**
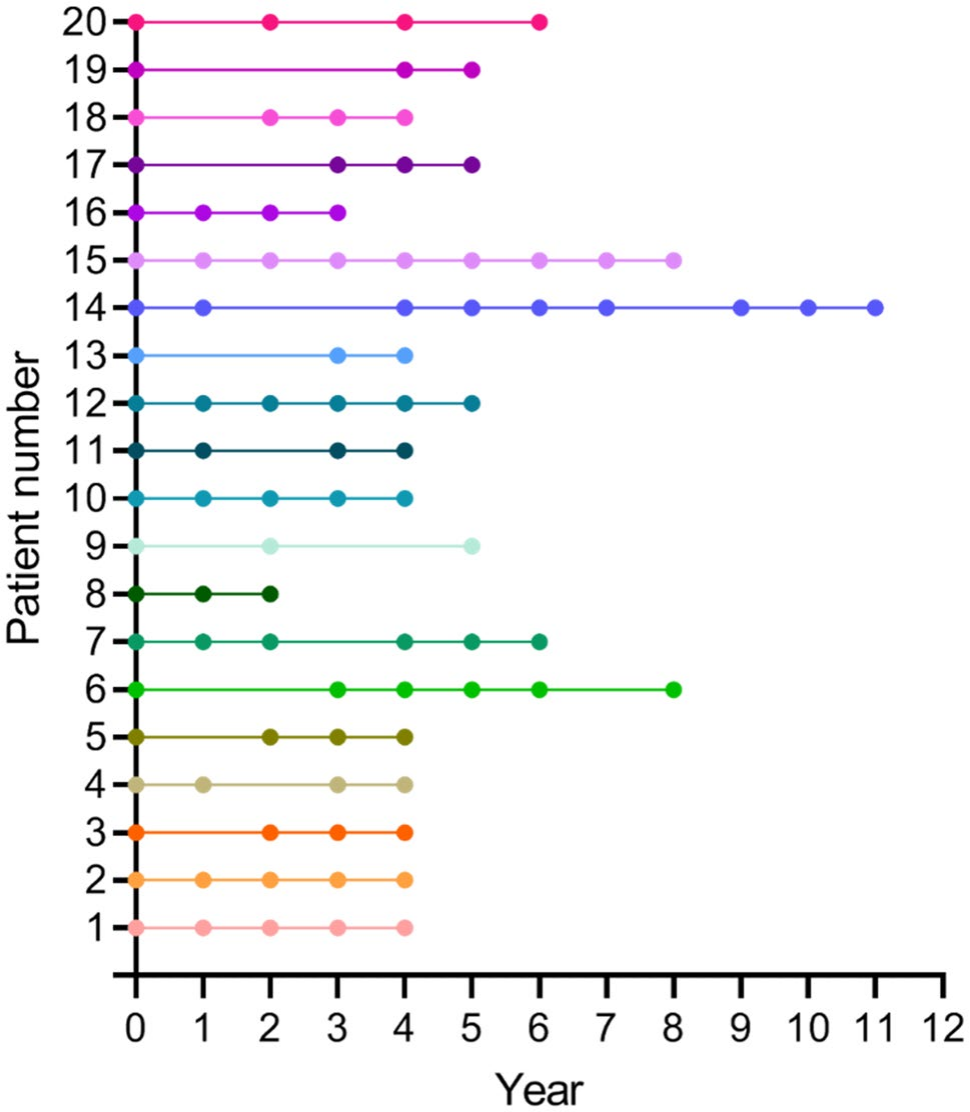
Sampling distribution for each MRSA-carrier (y-axis) per year (x-axis). Each dot represents a sample (n = 95).

**Figure 4:**
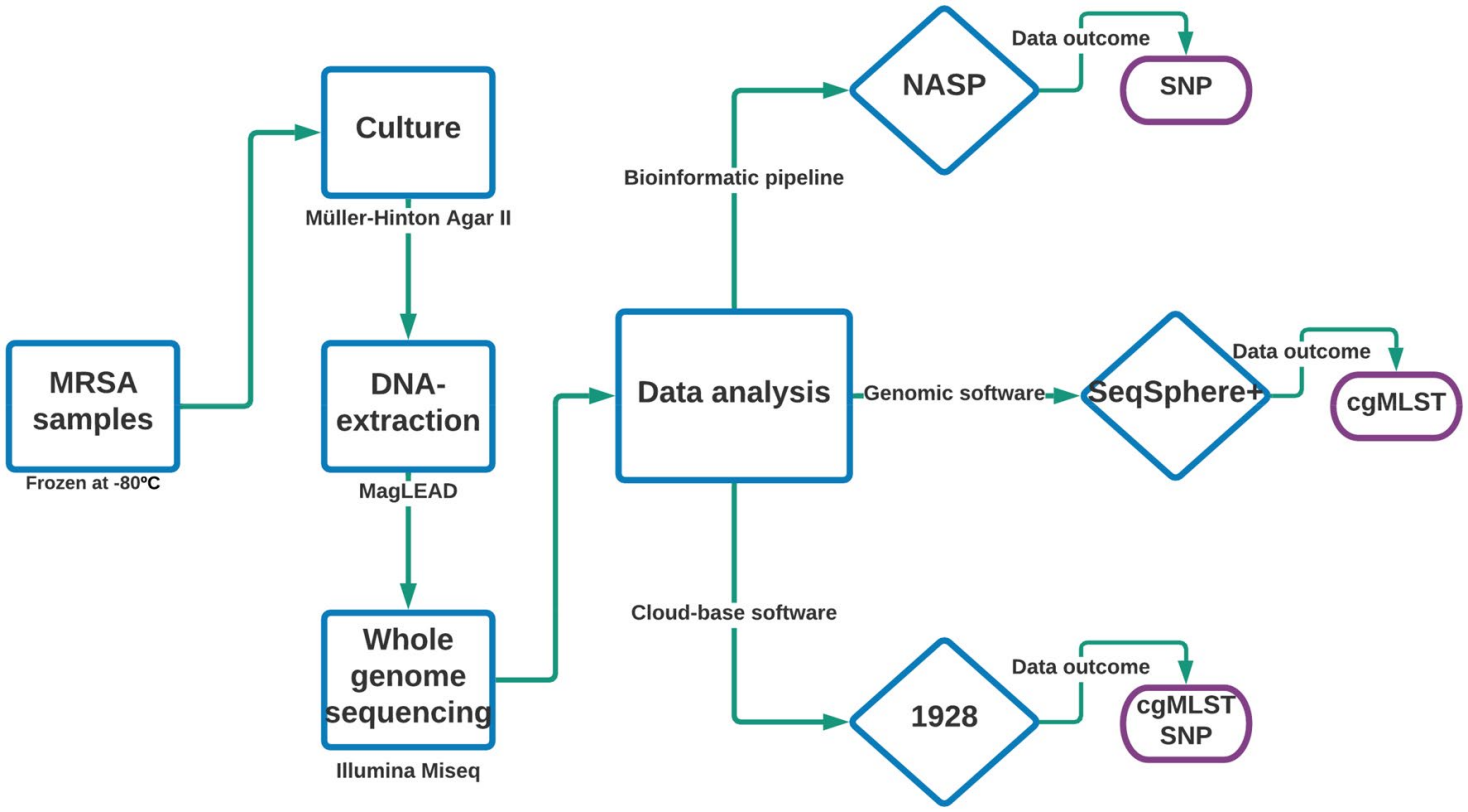
Flowchart for the analysis process from frozen MRSA samples to data analysis performed with different platforms for core genome multilocus sequence typing (cgMLST) and single nucleotide polymorphism (SNP).

Ninety-five MRSA isolates originating from twenty long-term carriers were collected from 2003 to 2019 by clinical routine and as part of the national monitoring program. One isolate per patient and per annual sampling (range 277 – 388 days (supplementary Table S1)) had been stored at − 80 °C in preservation medium (trypticase soy broth, BD, Diagnostic Systems, Sparks, MD, USA), supplemented with 0.3% (w/v) yeast extract (BD Diagnostic Systems) and 29% horse serum (SVA, Uppsala, Sweden) in clinical routine at the Department of Laboratory Medicine, Clinical Microbiology, at Örebro University Hospital, Örebro, Sweden. The number of samples (n = 95), obtained from the 20 long-term MRSA carriers, ranged from two to 9 samples per patient (Fig. 3) and the sample collection time frame ranged from two to 11 years (supplementary Table S2). A detailed flowchart for the entire analyse chain is shown in Fig. 4.

### Culture conditions and DNA extraction

MRSA isolates were subcultured on BD Mueller–Hinton II agar (Becton Dickinson and Company, Sparks, MD, USA) and species identity was verified by using Matrix Assisted Laser Desorption/Ionization Time of Flight (MALDI-TOF) using database Örebro20181107 / DB 7864 + SR taxonomy (Microflex LT, Bruker Daltonics Scandinavia AB, Solna, Sweden). For subsequent WGS, all isolates were incubated overnight on Mueller-Hinton Agar II (BD, Sparks, USA) at 36 °C. The isolates were then suspended in 200 µl 0.85% NaCl, pre-heated and shaken during one hour at 37 °C with lysozyme 72.000 U (Sigma L6876-1G) and Lysostaphin 135 U (Sigma, L9043, 5 MG). Genomic DNA was purified using the magLEAD 12gC, MagDEA Dx kit (ExScale, Chiba, Japan), following the manufacturer’s instructions. DNA was quantified using a Qubit fluorometer (Thermo Fisher, Waltham, MA). Purity 260/280 and 260/230 was measured using a NanoDrop 1000 (Thermofisher) and the fragmentation of the samples was determined using the DNA integrity number (DIN) by using a TapeStation 4200 system (Agilent, Santa Clara, CA).

### Library preparation and whole genome sequencing

Sequencing libraries were constructed using the Nextera XT DNA library preparation kit (Illumina, San Diego, CA, USA). Amplification of the tagmented DNA was performed using index primers, and the amplified products were purified with the ACSIA NGS^LibPrep^ Edition (PrimaDiag, Romainville, France), using AMPure XP beads (Beckman Coulter, Brea, CA). The normalization and pooling were performed manually, based on the size of each fragment determined using TapeStation and the DNA concentration was measured with Qubit. Sequencing was performed using an Illumina MiSeq Platform (Illumina, San Diego, CA) with MiSeq Reagent Kit v3, 600 cycle kit (Illumina) according to the manufacturer's instructions.

### Data analysis

The genome sequencing data of all isolates (n = 95) from the 20 long-term MRSA carriers were processed by the software Ridom SeqSphere+ 5.0.0 (Ridom GmbH, Münster, Germany), the cloud-based 1928 platform (1928 Diagnostics, Gothenburg, Sweden), and the Northern Arizona SNP Pipeline (NASP) version 1.0.1 for the cgMLST profiling and SNP analysis to evaluate the genomic variation rate (supplementary Table S2). A minimum coverage of more than 50-fold was considered acceptable quality for the SNP analysis. Two publicly available *S. aureus* reference genomes, COL (NC_002951.2)^29,30^ and Newman (NC_009641)^31^, were used for alignment of the samples in each method. The chosen references in this study are based on the default settings for each cgMLST approach, which are used in the clinical routine for NGS analysis at the microbiology laboratory at Örebro University hospital.

### cgMLST analysis and molecular typing by Ridom SeqSphere+

The reads were de novo assembled using Velvet (General public licence version 2.0) integrated in Ridom SeqSphere+^32^ using default settings: reads were trimmed until the average base quality of 30 was reached in a window of 20 bases. The samples were aligned to the COL reference sequence analysed by cgMLST using the software Ridom SeqSphere+ scheme based on 1861 genes^33,34^. More than 50-fold average coverage with an average read length of > 200 bp, and percentage good targets for cgMLST ≥ 97% was considered acceptable sequence quality for further data analysis. A phylogenetic tree was constructed in SeqSphere+ using their neighbour-joining tree algorithm (Fig. 1) and distance matrix calculation^35^ was performed; missing values were pairwise ignored. Additional information was extracted regarding the ST, CC, *spa* and CT. The CT is an additional identifier that clusters together samples with very similar cgMLST profiles and every combination of genomic allele profiles is given a unique identifier. The threshold used to define the CT in the cgMLST analysis was up to 24 allele differences (https://www.cgmlst.org/ncs/schema/141106/)^10^.

### cgMLST analysis by 1928 platform

For the 1928 platform, core gene data for all sequenced bacteria were extracted by a *k*-mer based allele calling method. The core genome scheme is based on 1704 genes built on the Newman reference genome^31^. More than 30-fold average coverage over the whole genome in combination with ≥ 95% of good cgMLST targets are considered acceptable sequence quality. The 1928 platform’s cgMLST method uses a custom developed allele-calling algorithm based on an alignment-free *k*-mer approach. Phylogenetic trees were constructed using 1928 UPGMA-tree algorithm and distance matrix calculations were made by calculating all pairwise allelic differences between samples, missing genes were pairwise ignored^36^.

### SNP analysis by 1928 platform

The 1928 platform’s SNP analysis pipeline uses Burrows-Wheeler aligner (v 0.7.17-r1188)^37,38^ for read alignment and FreeBayes (v1.3.2)^39^ for variant calling. The same publicly available reference genome for *S. aureus* were used as in the NASP analysis, COL and Newman. To account for putative recombinant regions, SNPs within a window of 25 base pairs were filtered out and excluded from the analysis. The window size was determined based on SNP density estimation over the genome. Distance matrices were computed from pairwise comparisons both including and excluding putative recombinant regions (supplementary Table S3).

### SNP analysis by NASP

The sequencing data was aligned to the COL and Newman reference genome using NASP v1.0.1^40^ to identify SNPs within the core genome. SNPs were called by aligning raw sequence reads with BWA (v 0.7.5a) and subsequent use of GATK (v 4.2.0.0) requiring at least tenfold sequencing depth and > 90% unambiguous base calls for all positions. Subsequently, the resulting SNP matrix files were used to obtain pairwise comparisons and create distance matrices.

### *spa* typing verified by Sanger sequencing

The analysis performed on the MRSA isolates by SeqSphere+ includes *spa* typing. However, not all the *spa* types for all the samples were successfully identified using WGS-data, therefore, spa typing for the isolates where the *spa* type had not been reported and additionally in the cases where the *spa* type differed was performed according to Berglund et al.^41^. The missing *spa* types were assigned using the software Ridom Staph (Ridom Gmbh, Würzburg, Germany)^42^.

### Statistical analysis

The average, mean, standard deviation, median and range, were performed with Statistical software SPSS version 25 to calculate the distance between samples for each patient and for the different analysis. The pairwise relatedness of cgMLST and SNP were calculated according to the allelic and SNP differences between samples, respectively.

## Supplementary Information

Supplementary Table S1.

Supplementary Table S2.

Supplementary Table S3.

## Data Availability

The data of this study are available by contacting the corresponding author upon reasonable request.
